# Melatonin Modulates Macrophage Polarization and Immunometabolic Responses in the Colostrum of Obese Mothers

**DOI:** 10.3390/metabo16060420

**Published:** 2026-06-15

**Authors:** Silvia Hannah Bilotti Ratto Gomes da Silva, Danielle Cristina Honorio França, Kênia Maria Resende Silva, Emanuelle Carolina Honorio França, Viviane Francelina Luz, Arce dos Santos Sfredo, Tassiane Cristina Morais, Eduardo Luzía França, Adenilda Cristina Honorio-França

**Affiliations:** 1 Institute of Biological and Health Science, Federal University of Mato Grosso, Barra do Garças 78600-000, MT, Brazil; silviahannahbrgs@gmail.com (S.H.B.R.G.d.S.); keniarezende1@gmail.com (K.M.R.S.); emanuellefranca57@gmail.com (E.C.H.F.); vivianefrancelina@gmail.com (V.F.L.); arcesfredo@gmail.com (A.d.S.S.); 2 Health Sciences Institute, Federal University of Mato Grosso, Sinop 78557-287, MT, Brazil; danielle.franca@ufmt.br; 3 School of Sciences, Santa Casa de Misericordia de Vitoria, EMESCAM, Vitoria 29045-402, ES, Brazil; morais.tassiane@gmail.com

**Keywords:** melatonin, obesity, colostrum, macrophages, cytokines, nitric oxide synthase

## Abstract

**Background/Objectives**: Obesity is a major public health problem associated with chronic inflammation and functional alterations in multiple organs and systems. Few studies have examined colostrum from obese mothers, particularly with respect to macrophage function, enzyme and cytokine concentrations, and the role of melatonin in immune modulation. This study aimed to evaluate melatonin levels and their effects on macrophage polarization, cytokine concentrations, nitric oxide synthase [iNOS], and arginase in colostrum from obese mothers. Colostrum samples were collected from eutrophic mothers [BMI: 18.5–24.9 kg/m^2^] and obese mothers [BMI: ≥30 kg/m^2^]. **Methods**: Macrophages were isolated by density gradient and treated with melatonin. The expression of M1 and M2 macrophages and cytokine concentrations were assessed by flow cytometry, while melatonin levels in colostrum supernatants, iNOS, and arginase in cell lysates were determined by ELISA. **Results**: An endogenous increase in melatonin was also observed in the colostrum of obese mothers. Maternal obesity has been shown to reduce M1 and M2 macrophage expression, increase nitric oxide synthase [NOS] activity, and elevate interleukin-6 [IL-6] and interleukin-17 [IL-17] levels. However, melatonin treatment restored M1 and M2 macrophage levels and reduced inducible nitric oxide synthase [iNOS] and arginase production to levels similar to those observed in mothers of healthy weight. **Conclusions**: these findings suggest that maternal obesity creates a pro-inflammatory environment in colostrum, characterized by altered macrophage polarization, altered cytokine secretion, and an imbalance in the enzymatic activities of iNOS and arginase within the L-arginine metabolic pathway. Both natural and supplemental melatonin exhibited immunomodulatory, antioxidant, and anti-inflammatory effects, helping to restore immune balance in colostrum. These results emphasize the potential benefits of melatonin as an immunometabolic modulator and its contribution to understanding immunometabolic regulation in obese mothers.

## 1. Introduction

Obesity is a multifactorial and complex condition resulting from the interaction between genetic, epigenetic, metabolic, behavioral, and socioeconomic factors [1,2]. In recent decades, its prevalence has increased significantly among women of reproductive age, contributing to the growing number of pregnancies associated with excess weight. Maternal obesity has also been associated with an increased risk of gestational complications, including preeclampsia, gestational diabetes mellitus, cesarean delivery, and fetal macrosomia, in addition to long-term metabolic and immunological repercussions for the child [3,4,5,6].

Obesity is defined as a condition of persistent low-grade inflammation, involving increased macrophage infiltration into adipose tissue and heightened production of pro-inflammatory cytokines [7,8]. This results in an inflammatory profile that alters macrophage phenotype. In this environment, M1-type macrophages are predominant, releasing reactive oxygen species, nitric oxide, and other pro-inflammatory mediators. During pregnancy, this inflammatory condition can negatively impact the intrauterine environment, potentially disrupting the maturation of the fetal immune system and increasing the newborn’s risk of developing inflammatory and metabolic disorders in the future [9,10,11].

The L-arginine metabolic pathway plays a crucial role in regulating the inflammatory response. This pathway is influenced by the competition between two enzymes: inducible nitric oxide synthase [iNOS] and arginase. iNOS is associated with M1-type macrophages and the production of nitric oxide [NO], which is an important cytotoxic and inflammatory mediator. In contrast, arginase—particularly the arginase-1 [ARG1] isoform—is associated with M2-type macrophages, which facilitate tissue repair, the resolution of inflammation, and cellular remodeling [12,13]. Therefore, the balance between iNOS and arginase serves as an important functional marker of macrophage polarization and immunometabolic regulation.

Under obesity conditions, alterations in arginase activity and NO bioavailability have been associated with insulin resistance, endothelial dysfunction, and the maintenance of systemic inflammation [14,15]. During pregnancy, these mechanisms become particularly relevant, since vascular and inflammatory alterations may directly affect the maternal-fetal interface and the immunological components transferred to the newborn.

Human colostrum is a major mediator of neonatal immunity, rich in immunoglobulins, cytokines, hormones, growth factors, and immunocompetent cells [6,16]. Among these cells, macrophages account for approximately 30–40% of the leukocytes in colostrum and play a fundamental role in neonatal immune defense due to their high phagocytic capacity and production of microbicidal metabolites [17,18,19]. However, evidence suggests that maternal obesity can modify the functional profile of these cells, favoring a pro-inflammatory microenvironment in colostrum characterized by alterations in macrophage polarization and inflammatory cytokine production [11,20].

Studies have indicated that melatonin represents an important immunometabolic modulator. In addition to its chronobiological function, melatonin exhibits antioxidant, anti-inflammatory, and immunoregulatory properties and is naturally present in human colostrum [17,21]. This hormone can modulate macrophage polarization by reducing the pro-inflammatory M1 profile and favoring the anti-inflammatory M2 phenotype, as well as regulating the expression of iNOS, arginase, and inflammatory cytokines [19,22,23].

Despite evidence of melatonin’s immunomodulatory effects, studies investigating its influence on macrophage polarization and the iNOS/arginase enzymes in the colostrum of mothers with obesity remain limited. Therefore, understanding the interactions among maternal obesity, systemic inflammation, and immunometabolic mechanisms in colostrum can help elucidate potential repercussions on neonatal immunity and contribute to a better understanding of the mechanisms underlying immune modulation during early life. Thus, this study aimed to evaluate the modulatory potential of melatonin on macrophage polarization, iNOS and arginase concentrations, and the cytokine profile in human colostrum from mothers with obesity.

## 2. Materials and Methods

### 2.1. Study Design and Participants

This cross-sectional study included 60 clinically healthy women recruited at the Municipal Hospital of Barra do Garças, Mato Grosso, Brazil, in 2025. Participants were allocated into two groups according to their pre-pregnancy body mass index [BMI]: eutrophic [18.5–24.9 kg/m^2^; n = 30] and obese [BMI ≥ 30 kg/m^2^; n = 30].

Eligibility criteria included maternal age between 18 and 35 years, documented pre-pregnancy weight or weight recorded up to the 13th week of gestation, delivery occurring between 37 and 41 6/7 weeks of gestation, negative screening for hepatitis, HIV, and syphilis, absence of dietary restrictions during pregnancy, and provision of written informed consent. Women with gestational diabetes mellitus, multiple pregnancies, fetal congenital anomalies, or preterm birth before 36 weeks of gestation were not eligible for inclusion in the study.

**Figure 1:**
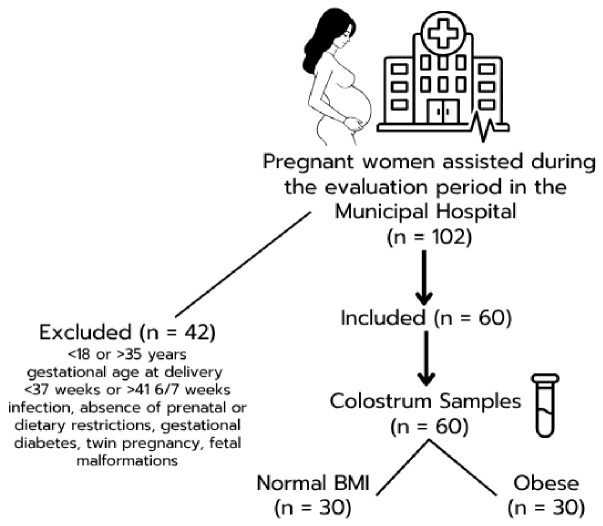
Representative scheme for obtaining samples.

Ethical considerations focused on the use of biological material for scientific purposes, ensuring the confidentiality of donors’ identities and the absence of coercion or conflicts of interest involving the institution or individuals participating in the study. Sample collection procedures followed the technical protocols established by the participating health services. All lactating women were informed of the study objectives in advance, and biological samples were collected and used only after written informed consent was obtained using an Informed Consent Form. The experimental design and procedures for colostrum sample collection are presented in Figure 1.

### 2.2. Colostrum Macrophage Isolation and Colostrum Supernatant

Approximately 8 mL of colostrum was collected from each participant. Samples were centrifuged at 160× *g* for 10 min at 4 °C, yielding three distinct phases: a cellular pellet [“cell button”], an intermediate aqueous phase, and an upper fat layer. The upper fat layer was discarded, and the supernatant was collected and stored, while the cellular pellet was preserved for further analyses. Cells were resuspended in Medium 199 culture medium [Gibco, Thermo Fisher Scientific (Grand Island, NY, USA] ] and subjected to Ficoll-Paque density gradient separation [Cytiva, Marlborough, MA, USA] for 40 min at 160× *g* at 4 °C. Cell counts were performed using a Neubauer chamber [(Labor Optik Ltd., Lancing, UK], and the cell concentration was adjusted to 2 × 10^6^ cells/mL. Cells were immediately used for functional assays, while the colostrum supernatant was stored at −80 °C for subsequent analyses.

### 2.3. Melatonin Hormone Quantification

Colostrum melatonin concentrations were determined by enzyme-linked immunosorbent assay [ELISA] using a commercial kit [Immuno-Biological Laboratories, IBL, Hamburg, Germany]. The assay detection limit was 1.6 pg/mL, with intra- and inter-assay coefficients of variation ranging from 3.0 to 11.4% and from 6.4 to 19.3%, respectively.

Before quantification, melatonin was extracted by affinity chromatography using standardized extraction columns. Columns were conditioned with methanol and double-distilled water, after which standards, controls, and samples were applied. Following washing with 10% methanol, melatonin was eluted with methanol, and the solvent was evaporated using a CentriVap SpeedVac concentrator [Kansas City, MO, USA]. The extracted material was then reconstituted in 0.15 mL of double-distilled water and immediately analyzed.

For ELISA, 50 μL of standards, controls, and samples were added to microplate wells, followed by melatonin-biotin and anti-melatonin antiserum. After incubation at 4 °C for 20 h, wells were washed and incubated with the enzyme conjugate for 120 min at room temperature. Subsequently, p-nitrophenyl phosphate [PNPP] substrate was added, and the reaction proceeded for 40 min under agitation. The reaction was terminated with a stop solution, and absorbance was measured at 405 nm using a microplate reader. Melatonin concentrations were calculated from the standard curve and expressed as pg/mL.

### 2.4. Melatonin Hormone Modulation

Colostrum macrophages were incubated with melatonin for 60 min. For each assay, control cells [2 × 10^6^ cells/mL] were incubated for the same period in Medium 199 in the absence of melatonin or cytokines, as specified in the experimental protocol. The final melatonin concentration was 100 ng/mL, as previously determined by Honorio-França et al. [17] and Fagundes et al. [24].

### 2.5. Immunophenotyping and Macrophage Identification and Polarization

Colostrum-derived cells were washed with phosphate-buffered saline [PBS] containing bovine serum albumin [BSA] and incubated for 10 min at 4 °C. Cells were then stained with 5 μL of FITC-conjugated anti-CD14 antibody, and a PE-conjugated IgG1 isotype control was used to evaluate nonspecific staining. Flow cytometric analysis was performed after exclusion of debris and non-cellular events based on forward scatter [FSC] and side scatter [SSC] parameters. The CD14+ population was gated and identified as macrophages, which were subsequently used for polarization analysis, as previously described by Benoit et al. [25].

For polarization assessment, cell suspensions were stained with antibodies against CD197, CD86, and CD163, followed by fixation and permeabilization using Cytofix/Cytoperm solution [BD Biosciences, San Jose, CA, USA]. Macrophages expressing CD197+CD86+ were classified as M1 phenotype, whereas CD14+CD163+ cells were classified as M2 phenotype.

Before sample acquisition, flow cytometer performance was verified according to the manufacturer’s recommendations using BD Calibrite™ 3 Beads. Fluorescence compensation was established using compensation beads and calculated with FACSComp™ software on Mac® OS 9 [BD Biosciences, San Jose, CA, USA]. The resulting compensation matrix was uniformly applied to all samples to ensure consistency and analytical reliability.

For each sample, a minimum of 10,000 events were acquired and analyzed using forward scatter [FSC], side scatter [SSC], and fluorescence intensity parameters. Data acquisition was performed on a FACSCalibur flow cytometer [BD Biosciences, San Jose, CA, USA] using CellQuest software version 7.5.3., and subsequent analyses were conducted with FlowJo version 7.2.5. The analytical procedure demonstrated good reproducibility, with a coefficient of variation [CV] of 4%.

### 2.6. Preparation of Cell Lysates

Colostrum mononuclear cells, treated or untreated with melatonin, were washed twice with phosphate-buffered saline [PBS] and resuspended in lysis buffer containing 1% Triton X-100. Cell lysis was performed by incubating cells for 5 min with gentle agitation to disrupt cellular membranes. After incubation, the lysates were clarified by centrifugation at 5000× *g* for 10 min at 4 °C to remove cellular debris. The resulting supernatants were collected and stored at −80 °C until subsequent analyses.

### 2.7. Nitric Oxide Synthase [NOS] Quantification

Nitric oxide synthase [NOS] concentrations were determined using a commercial ELISA kit [Cloud-Clone Corp., Wuhan, China], according to the manufacturer’s instructions. Colostrum supernatants and cellular lysates were previously prepared as described above. Briefly, 100 µL of samples, standards, or controls were added to microplate wells pre-coated with anti-NOS antibody and incubated at 37 °C for 60 min. After washing steps, 100 µL of biotinylated antibody and streptavidin–HRP conjugate were added, followed by incubation for 60 min at 37 °C. Subsequently, the wells were washed five times, and 100 µL of TMB substrate solution was added. The reaction was stopped with 2N sulfuric acid, and absorbance was measured at 450 nm using an ELISA Microplate Reader DR-200BS-NM-BI (Kasuaki, São Paulo, SP, Brazil). NOS concentrations were calculated from the standard curve and expressed as ng/mL.

### 2.8. Arginase Enzyme Quantification

Arginase concentrations were determined by enzyme-linked immunosorbent assay [ELISA] using a commercial kit [Cloud-Clone Corp., Wuhan, China], according to the manufacturer’s instructions. Colostrum supernatants and cellular lysates were previously prepared as described above. Briefly, 100 µL of samples, standards, or controls were added to microplate wells pre-coated with anti-arginase antibody and incubated at 37 °C for 60 min. After washing steps, 100 µL of specific biotinylated antibody and streptavidin–HRP conjugate were added, followed by incubation for 60 min at 37 °C. Subsequently, the wells were washed five times, and 100 µL of TMB substrate solution was added. The reaction was stopped with 2N sulfuric acid, and absorbance was measured at 450 nm using an ELISA Microplate Reader DR-200BS-NM-BI (Kasuaki, São Paulo, SP, Brazil). Arginase concentrations were calculated from the standard curve and expressed as ng/mL.

### 2.9. Cytokine Quantification in Human Colostrum Supernatants

The concentrations of IL-6, IL-10, TNF-α, and IL-17A in colostrum supernatants were determined using the Cytometric Bead Array [CBA] kit [BD Biosciences, USA]. Cytokine analyses were performed by flow cytometry on a FACSCalibur [BD Biosciences, San Jose, CA, USA], and data were analyzed with FCAP Array software version 3.0 [BD Biosciences, San Jose, CA, USA].

**Figure 2:**
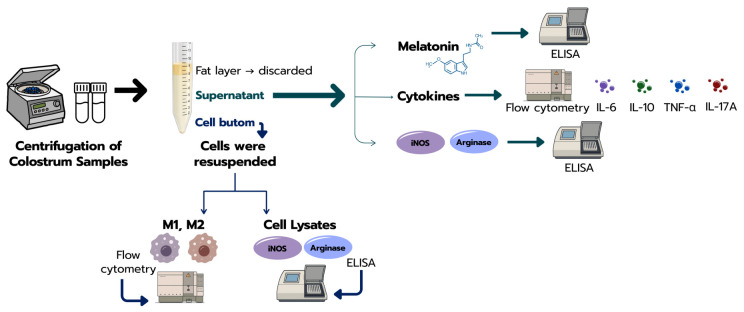
Experimental design of the study. Colostrum samples were fractionated by centrifugation. Melatonin, iNOS, and arginase were quantified by ELISA, while cytokines and macrophage polarization [M1/M2] were analyzed by flow cytometry.

The experimental design and sequence of analyses performed throughout the study are illustrated in Figure 2.

### 2.10. Statistical Analysis

Data were expressed as mean ± standard deviation [SD]. Data normality was assessed using the D’Agostino normality test. Statistical analyses of clinical data of mothers, melatonin concentrations, and cytokine levels were performed using Student’s *t*-test for independent samples. Macrophage polarization, iNOS, and arginase were evaluated using two-way ANOVA followed by Bonferroni’s post hoc test. Effect sizes were estimated using partial eta squared [η^2^p] for ANOVA analyses and Cohen’s d for pairwise comparisons. Data analysis was conducted using BioEstat^®^ version 5.0 [Mamirauá Institute, Belém, Brazil], with a 95% confidence level and a 5% significance level [*p* < 0.05].

## 3. Results

### 3.1. Clinical Data of Mothers

Table 1 presents the clinical and anthropometric characteristics of the participants’ mothers according to maternal nutritional status. Maternal mean age was similar between obese mothers [32.1 ± 5.2 years] and eutrophic mothers [30.5 ± 4.1 years]. Similarly, gestational age at delivery did not differ between groups; however, obese women exhibited a lower mean gestational age [37.7 ± 3.1 weeks] than eutrophic women [38.8 ± 2.8 weeks], but the difference was not statistically significant [*p* > 0.05].

Marked differences in body mass index [BMI] were observed throughout pregnancy. In the first trimester, eutrophic mothers had a mean BMI of 23.5 ± 2.4 kg/m^2^, whereas obese mothers had a substantially higher mean BMI of 34.5 ± 4.1 kg/m^2^ [*p* = 0.0245]. Similar differences were observed during the third trimester [41.5 ± 4.0 vs. 33.1 ± 3.9 kg/m^2^, respectively; *p* = 0.0123].

Fasting glucose levels were also elevated in the obese group [93.9 ± 7.1 mg/dL] compared with eutrophic mothers [83.9 ± 3.7 mg/dL; *p* = 0.0376], although within non-diabetic reference ranges. Hypertension was identified only among obese mothers, affecting 26% of participants, whereas no hypertensive cases were observed in the eutrophic group. No mothers from either group presented diabetes mellitus. The proportion of mothers reporting regular physical activity was similar between groups, corresponding to 43% in eutrophic women and 40% in obese women.

### 3.2. Melatonin Levels

**Figure 3:**
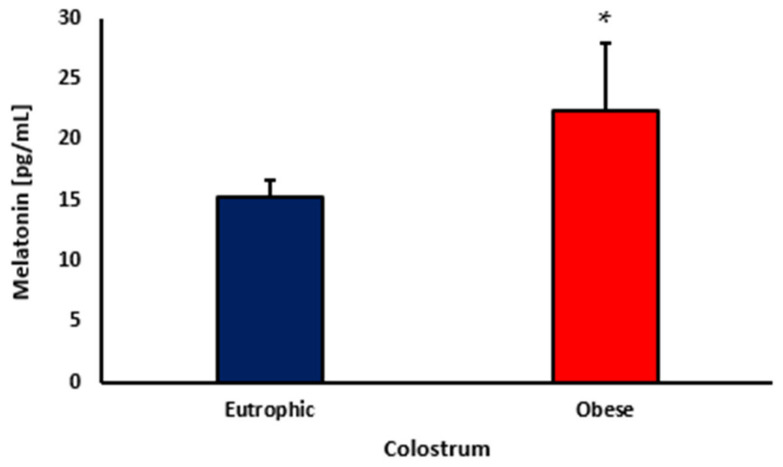
Melatonin concentration [pg/mL] in colostrum supernatants from obese mothers. * *p* < 0.042, indicating significant differences between eutrophic and obese mothers.

The melatonin concentrations [pg/mL] detected in colostrum supernatants from obese mothers are presented in Figure 3. A significant increase in melatonin levels [*p* = 0.042] was observed in the colostrum supernatant of obese mothers compared with eutrophic mothers.

### 3.3. Immunophenotyping of Macrophages

**Figure 4:**
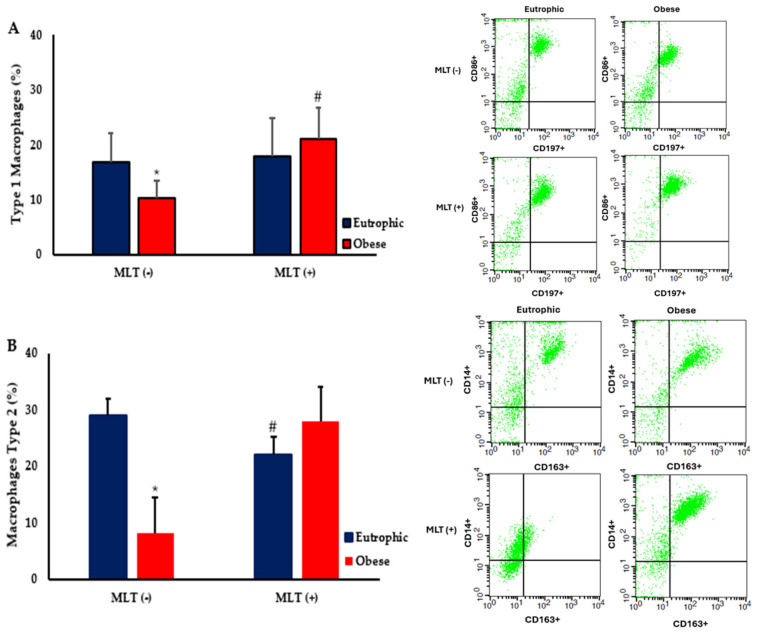
Expression of type 1 macrophages [M1, (**A**)] and type 2 macrophages [M2, (**B**)] in the colostrum of eutrophic and obese mothers in the presence or absence of melatonin. MLT [−], cells not treated with melatonin; MLT [+], cells treated with melatonin. * indicates differences between eutrophic and obese mothers under the same treatment condition [M1, *p* = 0.0185; M2, *p* = 0.0010]. # indicates differences between melatonin-treated and untreated samples within the same maternal group [M1, *p* = 0.0013; M2, *p* = 0.0318].

Figure 4 presents the expression of type 1 macrophages [M1—Figure 4A] and type 2 macrophages [M2—Figure 4B] in the colostrum of obese mothers, in the presence or absence of melatonin. Reduced expression of both M1 and M2 macrophages was observed in the colostrum of obese mothers compared with eutrophic mothers. Following melatonin treatment, the expression of both M1 and M2 macrophages increased in the colostrum of obese mothers, reaching levels similar to those observed in the colostrum of eutrophic mothers [Figure 4A,B].

### 3.4. Nitric Oxide Synthase [iNOS] Concentration

**Figure 5:**
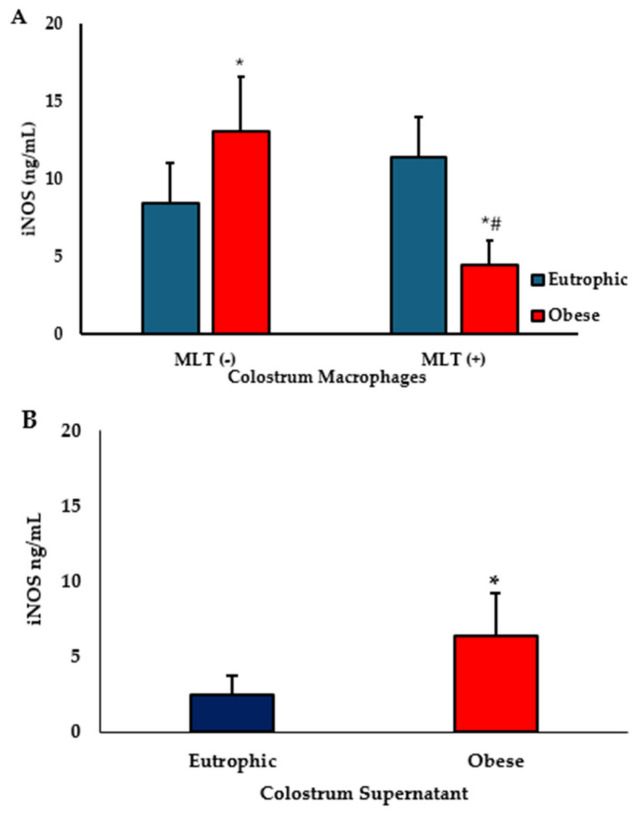
Nitric oxide synthase [iNOS] concentrations in macrophages in the presence or absence of melatonin (**A**) and in colostrum supernatants (**B**) from eutrophic and obese mothers. MLT [−], cells not treated with melatonin; MLT [+], cells treated with melatonin. In panel (**A**), * indicates significant differences between eutrophic and obese mothers under the same treatment condition [*p* = 0.0077], whereas # indicates significant differences between melatonin-treated and untreated macrophages within the same maternal group [*p* = 0.0021]. In panel (**B**), * indicates significant differences between eutrophic and obese colostrum supernatants [*p* = 0.0247].

Figure 5 presents the nitric oxide synthase [iNOS] concentrations in macrophages [Figure 5A] and in the colostrum supernatant [Figure 5B] of obese mothers. Increased iNOS concentrations were observed in colostrum macrophages from obese mothers compared with eutrophic mothers. Following melatonin treatment, iNOS concentration decreased in macrophages isolated from the colostrum of obese mothers. In addition, the colostrum supernatant of obese mothers showed increased NOS concentrations when compared with eutrophic mothers [Figure 5A,B].

### 3.5. Arginase Concentration

**Figure 6:**
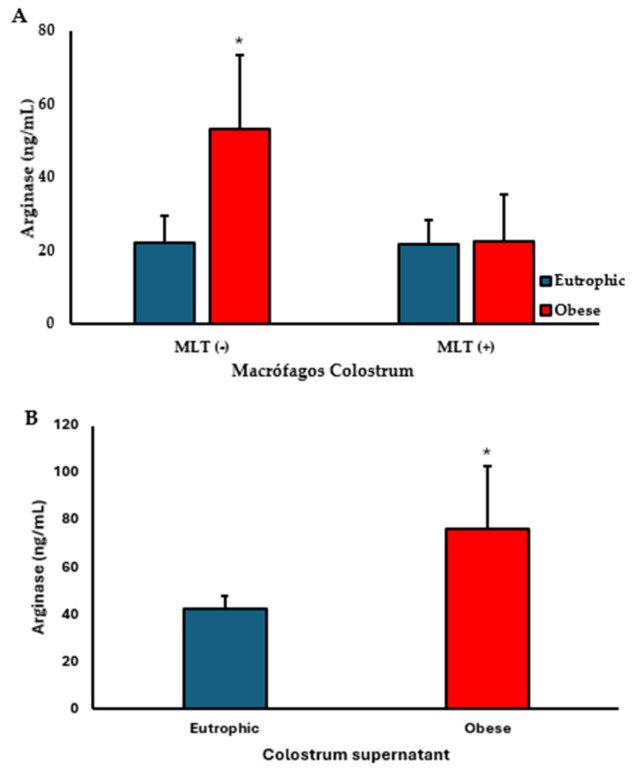
Arginase concentration [ng/mL] in macrophages in the presence or absence of melatonin (**A**) and in colostrum supernatants (**B**) from eutrophic and obese mothers. MLT [−], cells not treated with melatonin; MLT [+], cells treated with melatonin. In panel (**A**), * indicates significant differences between eutrophic and obese mothers under the same treatment condition [*p* = 0.0487]. In panel (**B**), * indicates significant differences between eutrophic and obese colostrum supernatants [*p* = 0.0462].

Figure 6 presents the arginase concentration in macrophages [Figure 6A] and colostrum supernatant [Figure 6B] from obese mothers. Increased arginase concentrations were observed in both macrophages and colostrum supernatant from obese mothers compared with eutrophic mothers. Melatonin treatment reduced the concentration of this enzyme in macrophages from obese mothers to levels similar to those observed in eutrophic mothers [Figure 6A].

### 3.6. Effect Size Analysis of Melatonin-Induced Changes in Macrophage Polarization and L-Arginine Metabolism

Effect size analyses were performed using partial eta squared [η^2^p] and Cohen’s d. Large-to-very large treatment effects were observed in macrophage polarization and enzyme activity. The η^2^p values were 0.260 for M1 macrophages, 0.793 for M2 macrophages, 0.618 for iNOS, and 0.468 for arginase.

The effect size analyses demonstrated distinct responses to melatonin according to maternal nutritional status. In eutrophic mothers, melatonin treatment produced negligible to small effects on M1 macrophages [d = 0.08], M2 macrophages [d = 0.27], and arginase activity [d = 0.08], and a small-to-moderate effect on iNOS levels [d = 0.40]. In contrast, in obese mothers, melatonin treatment was associated with large effects on M1 macrophages [d = 1.41], iNOS [d = 1.36], and arginase activity [d = 1.33], and an extremely large effect on M2 macrophages [d = 3.70].

### 3.7. Cytokines Levels

**Figure 7:**
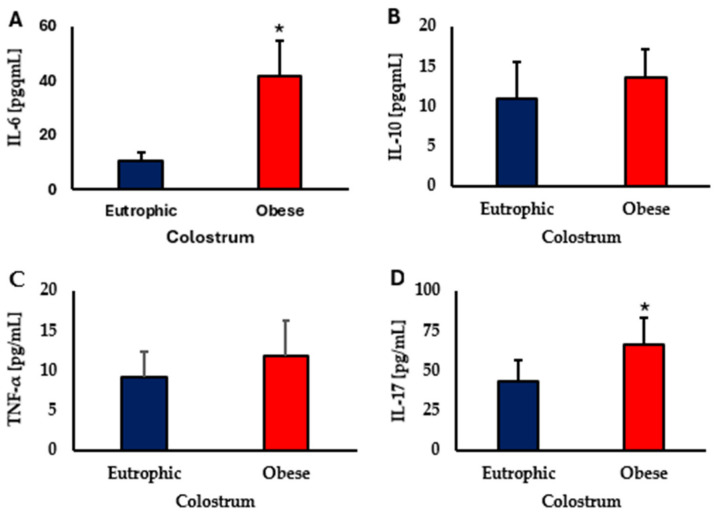
Cytokine concentrations [pg/mL] of IL-6 (**A**), IL-10 (**B**), TNF-α (**C**), and IL-17A (**D**) in colostrum samples from eutrophic and obese mothers. Significant difference between groups [*p* < 0.05]. Corresponding *p*-values were; IL-6 [*p* = 0.0212], IL-10 [*p* = 0.2140], TNF-α [*p* = 0.2069], and IL-17A [*p* = 0.0323]. * indicates significant differences between eutrophic and obese groups.

As shown in Figure 7, the concentrations of IL-6, IL-10, TNF-α, and IL-17A [pg/mL] were evaluated in colostrum samples. Among these cytokines, IL-6 and IL-17A were significantly increased in the colostrum supernatant of obese mothers compared with the eutrophic group.

**Figure 8:**
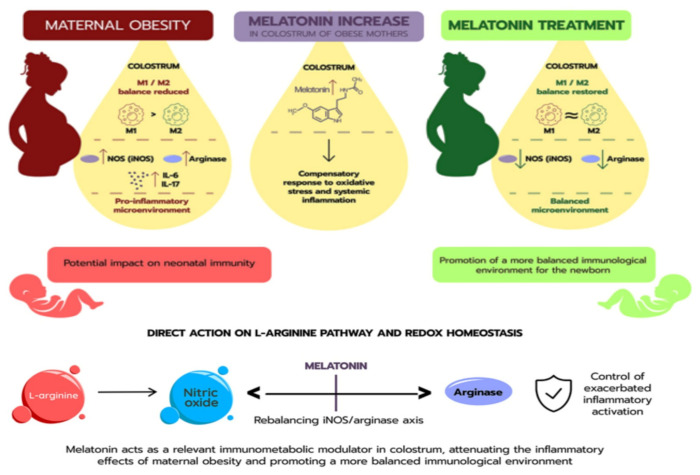
Schematic representation of the effects of maternal obesity and melatonin treatment on the immunological profile of colostrum.

Figure 8 summarizes the effects of maternal obesity on the immunological profile of colostrum and the modulatory role of melatonin. Maternal obesity induced a pro-inflammatory microenvironment characterized by an imbalance in macrophage polarization, elevated NOS and arginase levels, and elevated pro-inflammatory cytokine levels. In contrast, melatonin treatment restored immune balance and promoted redox homeostasis.

## 4. Discussion

The rise in global obesity among adults is reflected in the increased number of overweight women, and this may impact the health indicators of future generations [3]. Due to the rapid increase in the prevalence of maternal obesity during pregnancy worldwide, the adverse short- and long-term consequences for children of obese mothers represent a major public health risk in the 21st century [5]. Dietary and lifestyle interventions in women with obesity have suggested only limited beneficial effects [3,5]. This study observed alterations in the expression of type 1 and type 2 macrophages, melatonin levels, IL-6 cytokines, IL-17, NO synthase, and arginase in colostrum from mothers with obesity.

The results show that maternal obesity is associated with an unfavorable modulation of the immunological profile of colostrum, characterized by a reduction in the expression of type 1 [M1] and type 2 [M2] macrophages, suggesting an impairment of the immunoregulatory activity of phagocytic cells in the mammary microenvironment, which may affect the passive immunity conferred to the newborn.

The decrease in M1 macrophages in colostrum from obese mothers indicates a suppression of the local pro-inflammatory immune response, which is essential for the initial defense against pathogens. Under physiological conditions, M1 macrophages play a fundamental role in the production of inflammatory cytokines and the activation of microbicidal pathways mediated by nitric oxide and reactive oxygen species. However, obesity induces an immunometabolic dysfunction that alters macrophage polarization and impairs their effector capacity [7,26]. Although M1 macrophage expression was reduced, the elevated NOS levels suggest persistence of an activated inflammatory milieu, indicating that maternal obesity may induce a dysregulated macrophage phenotype rather than a classical suppression of inflammatory signaling.

The reduced expression of M2 macrophages in colostrum from obese mothers indicates impaired anti-inflammatory and reparative functions, consistent with the chronic low-grade inflammatory state associated with obesity. This result is particularly relevant because M2 macrophages contribute to tissue homeostasis and immune regulation, whereas obesity is characterized by a persistent inflammatory milieu that alters macrophage phenotype and function. The melatonin regulates macrophage polarization by suppressing M1-associated signaling pathways and enhancing M2-related markers through mechanisms involving NF-κB inhibition and STAT6 activation, thereby promoting an anti-inflammatory profile [22,27,28,29].

The present findings demonstrate that maternal obesity is associated with marked alterations in the immunometabolic composition of colostrum, characterized by increased concentrations of melatonin, NOS, and arginase in both the supernatant and colostrum-derived macrophages. These alterations support the concept that excess adiposity during pregnancy modulates the immune signals transferred to the newborn through interconnected inflammatory, metabolic, and redox-regulatory pathways.

The elevation of melatonin in the colostrum supernatant of obese mothers can represent a compensatory mechanism in the face of the systemic inflammatory state characteristic of this condition. Obesity is recognized as a state of chronic, low-grade inflammation, characterized by elevated levels of TNF-α and IL-6, as well as oxidative stress [7,8]. Melatonin has well-established antioxidant and immunomodulatory properties, acting as a direct scavenger of reactive species and regulator of inflammatory pathways, including NF-κB and NLRP3 [21,30]. In addition, its presence in human milk follows a circadian rhythm and plays an important role in the neonate’s immunological and metabolic maturation [31,32,33]. Thus, the observed increase may reflect an adaptive response of breast tissue to the maternal inflammatory environment.

Regarding nitric oxide synthase [iNOS] concentration, a significant increase in this enzyme was observed in both the supernatant and macrophages of colostrum from obese mothers. This increase reflects exacerbated inflammation and oxidative stress, since the inducible isoform of the enzyme [iNOS] is regulated by pro-inflammatory cytokines, such as TNF-α and IL-6, and its overexpression leads to excessive NO production and consequent cellular oxidative damage [34]. Higher iNOS expression is characteristic of M1 macrophage polarization and is associated with increased nitric oxide [NO] production and the production of pro-inflammatory mediators [13]. In adipose tissue from obese individuals, the iNOS/NO pathway is upregulated, contributing to insulin resistance, endothelial dysfunction, and the perpetuation of systemic inflammation [7,15]. The presence of this functional profile in colostrum suggests that maternal obesity may alter the phenotype of macrophages transferred to the neonate, potentially impacting neonatal immunometabolic programming.

Interestingly, treatment with melatonin significantly reduced NOS levels in macrophages from obese mothers, bringing them closer to those in the eutrophic group. This result is consistent with experimental evidence that melatonin inhibits iNOS expression and reduces NO production in activated macrophages, promoting a functional transition from the M1 phenotype to a less inflammatory profile [20,21]. This effect reinforces melatonin’s role as a modulator of macrophage polarization and the redox response in the colostrum environment.

Regarding arginase, a significant increase was observed in both the supernatant and lysate of macrophages from obese mothers. Arginase competes with iNOS for the common substrate L-arginine, diverting metabolism toward the production of L-ornithine and polyamines, associated with tissue repair, cell remodeling, and immune regulation [12,13]. Although classically associated with the M2 phenotype, increased arginase expression can coexist with elevated iNOS levels in chronic inflammatory contexts, reflecting a state of deregulated metabolic activation and competition for L-arginine [15]. The simultaneous increase in iNOS and arginase observed in obese mothers occurred despite the reduced frequencies of both M1 and M2 macrophage populations. This finding suggests that the immunometabolic alterations associated with maternal obesity may not be fully explained by the classical M1/M2 polarization paradigm. Instead, the data indicate a more complex state of macrophage metabolic dysregulation, characterized by concurrent activation of pathways involved in nitric oxide synthesis and L-arginine metabolism. Such a profile can reflect functional reprogramming of colostrum macrophages under obesogenic conditions, in which inflammatory and regulatory mechanisms coexist rather than representing mutually exclusive activation states. In obesity, this dysregulation of the L-arginine pathway has been linked to vascular dysfunction, reduced NO bioavailability, and systemic immunometabolic alterations [14].

Treatment with melatonin reduced arginase concentration in macrophages from obese mothers, normalizing it to levels observed in the eutrophic group. Although frequently associated with promoting the M2 phenotype, melatonin exerts regulatory effects that depend on the inflammatory microenvironment and may restore the balance between the iNOS and arginase pathways, thereby reestablishing L-arginine metabolic homeostasis [22,30,35]. Thus, the data suggest that melatonin modulates immunometabolic dynamics in colostrum, attenuating the exacerbated inflammatory activation induced by maternal obesity.

Maternal obesity was also associated with significant alterations in the colostrum cytokine profile. Increased concentrations of IL-6 and IL-17 were observed in the colostrum supernatant of obese mothers compared with eutrophic mothers, whereas IL-10 and TNF-α levels did not differ significantly between groups. Elevated IL-6 concentrations in colostrum from obese mothers may contribute to alterations in macrophage polarization and neonatal immune cell maturation. As a key mediator of acute and chronic inflammatory responses, increased IL-6 levels may also reflect the inflammatory milieu associated with maternal obesity [26,34].

The increase in IL-17 indicates activation of the Th17 pathway, associated with inflammatory and autoimmune responses. The elevated presence of this cytokine in colostrum suggests that maternal obesity promotes a pro-inflammatory microenvironment, which may contribute to immune imbalance in the infant [5].

On the other hand, the absence of significant changes in IL-10, an anti-inflammatory cytokine, suggests that the anti-inflammatory regulatory response may be insufficient to counterbalance the persistent inflammatory environment in the colostrum of obese mothers, indicating an imbalance between pro-inflammatory and anti-inflammatory responses that leads to a persistent inflammatory state [36].

In this study, the results demonstrate that maternal obesity is associated with substantial alterations in the immunological profile of colostrum, evidenced by reduced expression of M1 and M2 macrophage markers, increased nitric oxide synthase [NOS] activity, and elevated levels of the pro-inflammatory cytokines IL-6 and IL-17. These findings indicate that maternal obesity promotes a low-grade inflammatory environment in colostrum, which may influence the immune signals transferred to the newborn.

This effect is consistent with the antioxidant action of melatonin, which suppresses iNOS expression and reduces NO production under inflammatory conditions, and modulates iNOS transcription by inhibiting coactivators such as p300 and by negatively regulating transcription factor acetylation [21,22,27,37]. Regarding the cytokine profile, maternal obesity was associated with increased IL-6 and IL-17 levels, suggesting activation of pro-inflammatory pathways, including the Th17 pathway, whereas IL-10, an anti-inflammatory cytokine, remained unchanged. These findings reinforce the immunological imbalance in colostrum associated with obesity and indicate the relevance of inflammation-modulating mechanisms [34].

The effect size analyses further supported the biological relevance of the observed responses to melatonin. While melatonin produced only negligible to small effects on macrophage polarization and L-arginine metabolism-related markers in eutrophic mothers, substantially larger effects were observed in obese mothers. Particularly notable were the large effects on M1 macrophages, iNOS, and arginase activity, as well as the extremely large effect on M2 macrophages. These findings suggest that the immunometabolic alterations associated with maternal obesity may increase the responsiveness of colostrum macrophages to melatonin-mediated regulation, reinforcing the differential impact of melatonin on colostrum macrophages depending on maternal inflammatory and metabolic status.

The M1/M2 macrophage, NOS, and cytokine data demonstrate that maternal obesity is associated with alterations in multiple components of the colostrum immune system, including macrophage polarization and the production of inflammatory mediators. Under the experimental conditions evaluated, melatonin treatment attenuated these alterations, thereby modulating macrophage polarization, inflammatory responses, and the colostrum microenvironment. Thus, the present findings support a potential role for melatonin in regulating immunometabolic pathways associated with maternal obesity and contribute to a better understanding of the mechanisms underlying colostrum immune regulation [21,28,29,37].

This study has some limitations that should be considered. First, the relatively moderate sample size may limit the generalizability of the findings. In addition, the study population consisted of women from a specific region of Brazil, which may restrict the extrapolation of the results to other populations with different demographic, socioeconomic, and environmental characteristics. The cross-sectional design precludes establishing causal relationships between maternal obesity and the immunological changes observed in colostrum. Furthermore, only clinically healthy women without major comorbidities were included, which may limit the applicability of the findings to populations with more complex clinical profiles. Neonatal clinical outcomes were not evaluated, preventing assessment of the potential impact of these findings on infant health and development.

Additionally, the effects of melatonin were investigated in vitro, which may not fully capture the complexity of the colostrum microenvironment in vivo or predict its long-term effects beyond the immediate postpartum period. Finally, although the groups were characterized according to maternal nutritional status, other factors that may influence colostrum composition, such as dietary habits, lifestyle characteristics, and additional maternal metabolic variables, were not extensively evaluated. Therefore, future studies with larger cohorts, longitudinal follow-up, and evaluation of neonatal outcomes are needed further to elucidate the biological and clinical relevance of these findings.

## 5. Conclusions

Maternal obesity altered the immunological profile of colostrum, characterized by changes in M1 and M2 macrophage polarization, increased nitric oxide synthase [iNOS] and arginase concentrations, and elevated levels of pro-inflammatory cytokines, including IL-6 and IL-17. These findings indicate an altered inflammatory and immunometabolic environment in colostrum that may influence immune-related components transferred to the newborn.

Increased melatonin concentrations were also observed in the colostrum of obese mothers, possibly representing a compensatory response to oxidative stress and systemic inflammation. In vitro melatonin treatment was associated with restoration of M1 and M2 macrophage levels and a reduction in iNOS and arginase concentrations toward values observed in eutrophic mothers. These findings suggest that melatonin can help regulate the iNOS–arginase axis and modulate immunometabolic pathways involved in redox balance and inflammatory responses.

Overall, the results support an immunomodulatory role of melatonin in colostrum cells from obese mothers under experimental conditions. Additional research is necessary to understand the physiological significance of these findings. and to clarify their potential implications for maternal and neonatal health.

## Figures and Tables

**Table 1:** Clinical data of mothers with different glycemic and weight statuses.

Parameters	Eutrophic	Obese	*p*-Value
Age [years]	30.5 ± 4.1	32.1 ± 5.2	0.1769
Gestational age [weeks]	38.8 ± 2.8	37.7 ± 3.1	0.4352
BMI-1	23.5 ± 2.4	34.5 ± 4.1	0.0245
BMI-2	33.1 ± 3.9	41.5 ± 4.0	0.0123
Fasting glucose	83.9 ± 3.7	93.9 ± 7.1	0.0376
Hypertension [%]	0	7 [26%]	____
Diabetes [%]	0	0	____
Physical activity [%]	13 [43%]	12 [40%]	____

Notes: Data are presented as mean ± SD or percentages, as appropriate. Significant differences between eutrophic and obese mothers as determined by Student’s *t*-test [*p* < 0.05]. BMI-1, body mass index in the first trimester [kg/m^2^]; BMI-2, body mass index in the third trimester [kg/m^2^]. Hypertension was defined as clinically diagnosed chronic or gestational hypertension. Physical exercise was defined as regular moderate physical activity performed at least three times per week.

## Data Availability

The data supporting the findings of this study are available from the corresponding authors upon reasonable request.

## Abbreviations

The following abbreviations are used in this manuscript:

ARG1	arginase-1
BMI	body mass index
CD	Cluster of Differentiation
ELISA	enzyme-linked immunosorbent assay
IL	interleukin
iNOS	inducible nitric oxide synthase
M1	macrophage type 1
M2	macrophage type 2
MLT	melatonin
NF-κB	nuclear factor kappa B
NLRP3	NOD-like receptor family pyrin domain-containing 3
NO	nitric oxide
NOS	nitric oxide synthase
PNPP	p-nitrophenyl phosphate
SD	standard deviation
STAT6	signal transducer and activator of transcription 6
